# Ultrasonic Evaluation of the Achilles Tendon in Patients Treated for Congenital Clubfoot: Comparison between Patients Treated with Plaster Alone, Achilles Tenotomy, and Z-Plasty Lengthening

**DOI:** 10.3390/children11050580

**Published:** 2024-05-11

**Authors:** Luisella Pedrotti, Barbara Bertani, Gabriella Tuvo, Redento Mora, Fabrizio Nasi, Federica Manzoni, Luca Marin, Francesco Moro, Federica De Rosa

**Affiliations:** 1 Locomotor System Diseases Unit, Department of Clinical Surgical, Diagnostic and Pediatric Sciences, University of Pavia, 27100 Pavia, Italy; 2 Orthopedic and Traumatology Unit, Città di Pavia Institute, 27100 Pavia, Italy; barbara.bertani@grupposandonato.it (B.B.); gabriella.tuvo@grupposandonato.it (G.T.); redento.mora@grupposandonato.it (R.M.); 3 Outpatient Ultrasound Service, Città di Pavia Institute, 27100 Pavia, Italy; fabrizio.nasi@libero.it; 4 Epidemiology Unit, Health Protection Agency of Pavia (ATS Pavia) Italy, 27100 Pavia, Italy; federica_manzoni@ats-pavia.it; 5 Laboratory of Adapted Motor Activity, Department of Public Health, Experimental and Forensic Medicine, University of Pavia, 27100 Pavia, Italy; luca.marin@unipv.it; 6 Department of Biomedical Surgical and Dental Sciences, University of Milan, Via Festa del Perdono 7, 20122 Milan, Italy; francesco.moro@unimi.it; 7 Pediatric Orthopedic and Traumatology Unit, Children’s Hospital, AON SS Antonio e Biagio e Cesare Arrigo, 15121 Alessandria, Italy; federica.derosa@ospedale.al.it

**Keywords:** ultrasound, congenital clubfoot, tenotomy, subcutaneous rupture

## Abstract

Background: Clubfoot is a common congenital deformity. The Ponseti technique, involving early corrective manipulations followed by applying long leg casts and Achilles tenotomy, is widely accepted as the preferred treatment. Rapid tendon healing after surgery has been documented, but the aspect regarding long-term tendon structure and properties is not known. Three cases of Achilles tendon rupture in adolescents previously treated for clubfoot have been described in the literature. As rupture is a rare event in this age group, a possible correlation with previous surgery has been hypothesized. The primary aim of the study was to compare the ultrasound findings of the Achilles tendon in patients treated for clubfoot, between patients treated with casting alone and with patients who underwent surgery (percutaneous tenotomy or Z-plasty lengthening). Methods: There were 22 asymptomatic patients (34 feet) with a median age of 12 years, previously treated for clubfoot, that were recruited for this study; the patients underwent an Achilles tendon ultrasound examination during a follow-up outpatient visit. Results: A greater thickness and increased number of structural alterations with the presence of hypoechoic areas of the operated tendons compared with those treated with plaster alone were observed (*p*-value: 0.0498 and <0.001, respectively). These ultrasound findings were indicative of tendon suffering, as seen in tendinopathies. Conclusions: The presence of ultrasound alterations in asymptomatic patients operated on for clubfoot requires careful control of the extrinsic factors of tendinopathy in order to reduce the risk of subcutaneous rupture.

## 1. Introduction

Clubfoot is a common congenital deformity, with a prevalence ranging from 1 to 6 per 1000 live births [1,2,3,4]. Because of frequent relapses, it is considered a difficult disease to treat [5,6]. It is commonly treated with the Ponseti method, which involves manipulations followed by plaster casts and, often, tenotomy of the Achilles tendon. The obtained correction is maintained with a night brace until the age of 4–5 years [7,8,9]. Achilles tendon tenotomy is a minimally invasive procedure, and it is performed in a variable percentage of cases, up to 85% [8,10,11]. In the event of a recurrence, the plaster cast and Achilles tenotomy procedure are repeated in ambulatory patients by percutaneous lengthening [12,13]. Z-plasty lengthening of the tendon is performed both in “complex” feet, and neglected or relapsed feet [10]. Achilles tenotomy or Achilles tendon lengthening are, therefore, essential procedures to obtain good results in congenital clubfoot treatment. The results of Achilles tenotomies have been extensively studied in terms of healing times. Many authors have shown complete restoration of the continuity of the tendon fibers 3–6 weeks after surgery [14,15,16,17] and good functional recovery with no restrictions in sports performance or activity [18,19]. The natural history of tendon healing after tenotomy or Z-plasty lengthening in clubfoot is not well understood [20]. As far as we know, the healing process of the Achilles tendon after lengthening at the myotendinous junction has been reported in CP [21]. Tendon features after Z-plasty lengthening have been described in rats, but we found no data in humans [22].

Three cases of Achilles tendon rupture in adolescents previously treated for clubfoot have been described in the literature. Since rupture is a rare event in this age group (globally the cases described are 5) a possible correlation with previous surgery has been hypothesized [23,24,25]. The primary aim of the present study was to compare the ultrasound findings of the Achilles tendon among patients previously treated for congenital clubfoot, between patients who were treated with casting alone and the ones patients who underwent surgery (percutaneous tenotomy or Z-plasty lengthening). The secondary aim was to carry out a descriptive analysis of the ultrasound parameters detected both on tendons undergoing tenotomy and those undergoing Z-plasty, with or without prior tenotomy.

## 2. Patients and Methods

### 2.1. Study Design

This is a cross-sectional observational study.

### 2.2. Setting and Time

Over an 8-month period (October 2021–May 2022), 22 patients (8 girls and 14 boys) previously treated for clubfoot were recruited for this study. The patients underwent an Achilles tendon ultrasound examination during a follow-up outpatient visit, at the Pediatric Orthopedics Clinic of Città di Pavia Institute in Pavia, Italy.

### 2.3. Population

In total, 22 patients (14 males and 8 females) were included in the present study, with a total number of 34 tendons. The median age value at the time of the survey was 12.0 years (IQR 8.0–16.0). The deformity was unilateral in 10 patients and bilateral in the remaining 12, for a total of 34 pathological feet. We divided the feet and tendons into 3 groups: Pirani score 1–2 (2 feet), Pirani score 3–4 (11 feet), and Pirani score 5–6 (feet).

Achilles tendon surgery was performed in 20 cases (11 patients underwent exclusively percutaneous tenotomies, 9 subsequently underwent lengthening with Z-plasty) and the mean interval since surgery was 9.8 years (SD 4.5 years). In 14 cases, the corrections were obtained with plaster cast alone.

### 2.4. Variables and Endpoints

Demographical data and clinical information (regarding the type of treatment and the type of surgery) were collected.

Ultrasound measurements of the Achilles tendon were reported.

The primary endpoint was the tendon thickness (measurement expressed in mm) in a neutral position. Secondary endpoints were as follows: the tendon thickness in a dorsiflexed position, the presence of vascularization, hypoechoic areas, and respective calcifications. Finally, in the unilateral forms, we compared the ultrasound findings of the healthy feet with those of the pathological side.

### 2.5. Data Sources and Data Collection

The examination was carried out by the same operator with a Hitachi Arietta 65 ultrasound machine (Fujifilm Italia S.p.A., Cernusco Sul Naviglio, (MI), Italy) using a linear probe. Because tissues with a fibrillar structure, such as tendons, have reduced echogenicity based on the angle of the transducer, attention was paid to the correct positioning of the probe in order to avoid mistakes in the interpretation of pathological defects. Patients lay in a prone position with their ankles extending over the edge of the table. Each foot was studied both in the neutral and dorsiflexion positions. Tendon images were viewed in real-time in longitudinal and transverse planes; the section site was identified as 2.0 cm above the calcaneal insertion. Diffuse or localized hypoechoic areas in the superficial layer of the tendon near the perithenonium, and the presence of calcifications were sought. Areas of vascularization and the number of vessels present were evaluated by means of color Doppler ultrasound.

### 2.6. Sample Size Considerations

The primary goal of the present study is to compare the ultrasound features of the Achilles tendons between the ones conservatively treated and the ones who underwent surgery. The primary endpoint was the tendon thickness in the neutral position. Variation of the tendon diameters in dynamic, found during the dorsiflexion maneuver, could be explained by the possibility that the measures would change in relation to the elasticity of the tendon itself.

Agarwal et al. [14] performed an ultrasound examination on 37 Achilles tendons (in 26 patients suffering from congenital clubfoot, with a mean age of 17 weeks and who underwent a tenotomy intervention). The mean Achilles tendon thickness was 2.52 mm before tenotomy, 3.82 mm three weeks after the tenotomy, 3.77 mm six weeks after surgery, and 4.03 at 1 year of age. From the 6th to the 12th month after surgery, a thickening of the Achilles tendon was observed. It, therefore, seems that, over time, the thickness of the tendon subjected to tenotomy tends to increase. Actually, the natural history of tendons subjected to tenotomy is not yet well known.

### 2.7. Sample Size Calculation

Considering a mean value of the Achilles tendon thickness in a neutral position equal to 4.9 mm (ds = 1.24) for the group of tendons with a congenital clubfoot that underwent surgery and a mean value of 3.7 mm (ds = 0.71) for the group of tendons with congenital clubfoot conservatively treated, a total number of 34 tendons with CCF, of which 20 treated surgically and 14 treated conservatively, allowed for estimating a mean difference of 1.27 mm between the average values of the two groups with the Student t test, ensuring a power > 90% and an alpha error equal to 0.05.

### 2.8. Statistics

The categorical variables were expressed as counts and percentages, and the continuous variables were described with mean and standard deviation and with median and interquartile range (IQR: 25th–75th percentile). In this way, a complete description of the data was provided, including mean and median as measures of central tendency, with the mean being affected by extreme values (outliers) and the median not being affected; standard deviation and IQR were reported as measures of dispersion. The normality of the distribution was checked with the Shapiro–Wilk test.

The comparisons between two groups for continuous variables were performed with the Mann–Whitney non-parametrical test, given the non-parametrical distribution of the data. Associations between categorical variables were studied with the Pearson’s χ^2^ or with the Fisher’s exact test, depending on the frequency distribution of the contingency table.

All of the tests were 2-sided; the significance level was set at α  =  0.05. A *p*-value  <  0.05 was considered statistically significant. Data analysis was performed using the STATA statistical package (version 17 or later; Stata Corporation, 2009, College Station, TX, USA).

## 3. Results

The results are summarized in Table 1.

The average tendon thickness was greater in the surgically treated feet than in those treated with casting alone, with a statistically significant difference both in the neutral position and in dorsiflexion (*p*-value 0.0498 and *p*-value 0.0013, respectively). In the tendons treated with casting alone, there was an average thickness reduction of 9.73% in the dorsiflexed position compared the neutral position, higher than that of surgically treated tendons (1.81% and 0% in tenotomy and Z-plasty lengthening, respectively); this means that the tendon subjected to stretching alone remained more elastic than the operated one, with a percentage of reduction in thickness in dorsiflexion that was very similar to that of the healthy tendon (10.67%). We cannot comment on the intrinsic elasticity of the tendons, which varied from subject to subject and is related to the severity of the disease; through the ANOVA test, we verified that the initial Pirani score did not statistically significantly influence the results. The absence of a direct relationship with the initial Pirani score led us to attribute greater importance to the type of treatment performed.

The vessels were observed on a Doppler ultrasound in 2 tendons among the operated feet; none were present in the conservatively treated feet. An insertional calcification was observed in one of the healthy control tendons, whereas no calcification was present in the surgically treated tendons. We have no explanation for this isolated observation.

The irregularity of the tendon structure with hypoechoic areas in the superficial tendon layers was observed in 16 surgically treated and 2 conservatively treated tendons, with a statistically significant difference (*p*-value < 0.001).

The comparison between tendons undergoing tenotomy and those undergoing Z-plasty, with or without prior tenotomy, showed no statistically significant differences in tendon thickness, both in neutral and dorsiflexed positions. However, the size was greater in the tenotomized tendons. We were not able to provide a clear explanation, but we hypothesize that, because tenotomy was performed earlier than Z-plasty, the newly formed repair tissue may be more exuberant.

In summary, the operated tendons had a greater thickness and a less homogeneous structure compared with the tendons treated with stretching alone; furthermore, the thickness reduction in dorsiflexion was greater in conservatively treated tendons, similar to what was observed in the healthy tendons.

## 4. Discussion

A surgical procedure on the Achilles tendon (tenotomy or Z-lengthening) is an integral part of the treatment of clubfoot [8,9,12,13]. We investigated ultrasound parameters in the Achilles tendons of asymptomatic patients treated in the past for congenital clubfoot. Statistically significant observed data were a greater thickness of the operated tendons compared with those treated with plaster alone (with no difference between the tenotomized ones and those with Z-plasty). The operated tendons also showed structural alterations, with a greater presence of hypoechoic areas. Our results were different from those observed by other authors, who documented tendon healing within a few weeks after tenotomy, observing minimal ultrasound anomalies during follow-up that did not seem to affect function [14,17,24,25]. The authors, however, reported data at a shorter distance from the operation, at 6 and 4 weeks, respectively [14,26]. The ultrasonographic characteristics found in our patients were similar to those observed in the case of tendinopathy, even in the absence of subjective symptoms; we looked for a possible explanation of these data. The healing process after a surgical procedure on a tendon can be compared to the tendon repair process after an injury. Tendon healing occurs in three overlapping phases: inflammatory phase, proliferative phase, and remodeling phase. During those stages, the repair tissue undergoes numerous changes, but never reaches the characteristics of healthy tendon [27,28,29].

Watzl analyzed the various phases of tendon healing by ultrasound examination in patients treated with tenotomy for congenital clubfoot; the author confirmed the ultrasound changes of the tendon structure that corresponded to those described during the biological healing process [15]. The healing mechanism after Z-plasty lenghtening was probably different, but we assumed that, in this case, the mechanical properties of the tendon were also not comparable to those of the healthy tendon. Weigl et al. showed the ultrasound aspects of healing after lengthening of the Achilles tendon at the neuromuscular junction in patients suffering from CP, with a short follow up (5.2 months) [20].

Kim et al. illustrated a Z-lengthening technique modification: they focused the discussion on cosmetic improvement and a reduction in complications such as overcorrection or recurrence [30].

As far as we know, the only data on the histopathological evaluation after achilloplasty refer to experimental work on rats, in which the tendon healing process has been analyzed in relation to the type of suture performed [21].

There are no certain data on the natural history of the tendon healing process after clubfoot surgery. Few cases of subcutaneous rupture of the Achilles tendon in adolescents undergoing Achilles tenotomy for clubfoot are described in the literature. In adolescence there are usually no intrinsic factors predisposing tendinopathy, so tendon alterations underlying subcutaneous rupture could be attributed to the previous surgery, as hypothesized by other authors [31,32].

**Figure 1:**
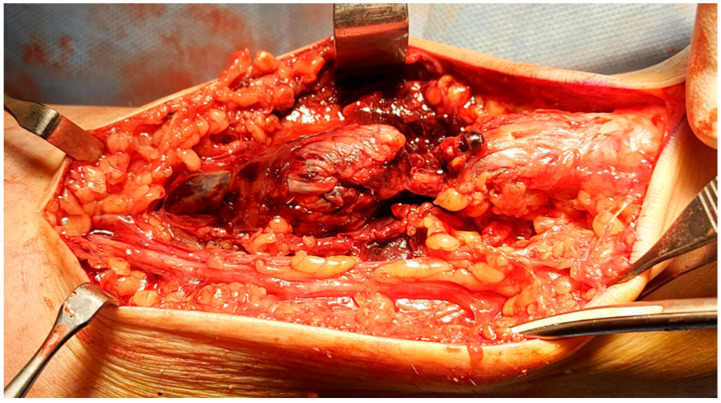
The intraoperative finding shows complete interruption of the Achilles tendon 2 cm at the calcaneal insertion. In addition to the areas of hemorrhagic infarction, there is a clear alteration of the macrostructure of the tendon.

**Figure 2:**
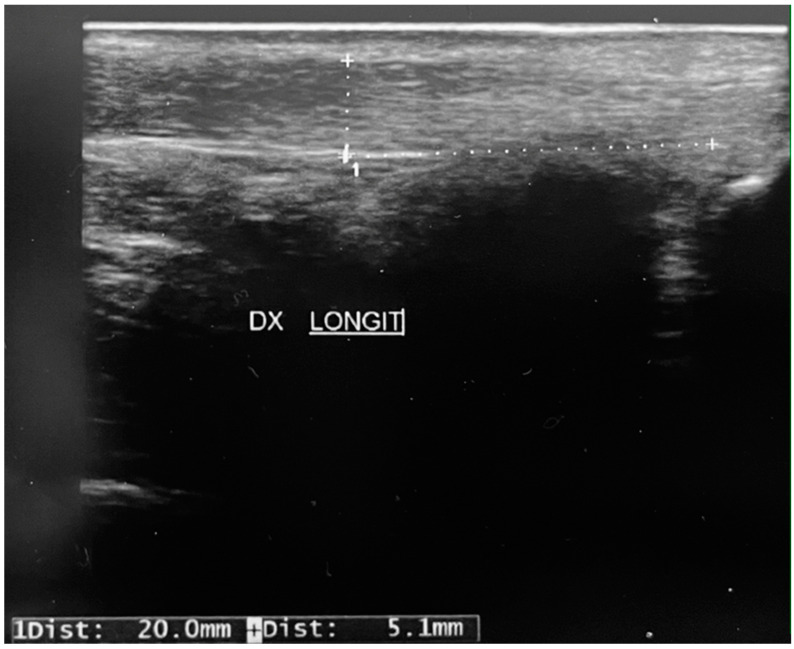
The ultrasound finding shows a thickening of the Achilles tendon and diffusely hypoechoic superficial areas 2 cm from the insertion on the calcaneus.

During data collection, one of the patients analyzed in our study suffered a rupture of the right Achilles tendon as she walked (Figure 1). The patient was a volleyball player and she was in good health. She had not taken drugs with a potential negative effect on tendons, such as quinolones or corticosteroids, and she had no extrinsic risk factors for tendinopathy, such as use of inappropriate footwear or overtraining. The ultrasound we previously carried out showed thickening of the Achilles tendon and diffusely hypoechoic superficial areas (Figure 2) 2 cm from the insertion on the calcaneus.

It seems interesting to underline that, in our patient, the finding of the ultrasound alterations corresponding to the site of the rupture could confirm the hypotheses of other authors. The hypothesis could be that the surgical approach to tendon retraction in clubfoot (whether by tenotomy or Z-plasty) is followed by a healing process that restores the continuity of the tendon. However, this process is characterized by haphazard proliferation of abnormal tenocytes, disruption of collagen fibers, and a subsequent increase in the non-collagenous matrix, as observed in tendinopathies [33]. These alterations could represent the substrate on which the sporting overload becomes decisive for subcutaneous rupture.

A limitation of this study is represented by the small sample size. Furthermore, the case series is heterogeneous in terms of the age and interval since surgery. This means that some of the data considered, such as the thickness of the tendon, may be variable in relation to age. However, we observed that only one value differed significantly from the others; almost the all of the patients, despite the age differences, appeared rather homogeneous.

The small number of patients did not allow us to form groups based on the type of activity practiced or its frequency; it would be interesting to study the characteristics of the tendon in relation to the mechanical load to which it is subjected.

The tendon tissue that suffered subcutaneous rupture was not submitted for histological examination; this was a mistake, as the comparison between the ultrasound and anatomopathological data could have offered ideas for discussion. To draw certain conclusions, a larger number of patients would be desirable.

## 5. Conclusions

Clubfoot is a relatively common congenital pathology. Complete correction of the deformity often requires a surgical procedure on the Achilles tendon. Some cases of subcutaneous tendon rupture have been described in these patients. Ultrasound has proven to be a sensitive technique for documenting tendon alterations, even in asymptomatic patients; it has been shown that in the presence of these alterations, there is a risk of developing tendinopathy, which is a predisposing factor for subcutaneous rupture. Ultrasound monitoring of patients treated for clubfoot and who practice intense sport could allow for the identification of risk situations, which require training adjustments or other preventive regimens in order to reduce the risk of rupture.

## Figures and Tables

**Table 1:** Ultrasound evaluation results.

Tendon Measurements	Healthy Tendon (10)	Casting Alone (14)	Tenotomy (11)	Z-Plasty Lenghtening (9)
**Tendon thickness in neutral position (mm)**	Mean (sd): 4.15 (0.87) Median (iqr): 4.1 (3.8; 4.8)	Mean (sd): 4.16 (0.91) Median (iqr): 4.2 (3.6; 4.7)	Mean (sd): 5.24 (1.12) Median (iqr): 5.3 (4.2; 5.9)	Mean (sd): 4.66 (1.16) Median (iqr): 4.5 (4.2; 4.8)
**Tendon thickness in dorsiflexion position (mm)**	Mean (sd): 3.87 (0.86) Median (iqr): 3.85 (3.4; 4.3)	Mean (sd): 3.71 (0.71) Median (iqr): 3.75 (3.4; 4.1)	Mean (sd): 5.34 (1.40) Median (iqr): 5.1 (4.5; 6.0)	Mean (sd): 4.56 (0.89) Median (iqr): 4.6 (4.1; 5.5)
**Average difference between tendon thickness in neutral position and in dorsiflexion, in percentage**	Mean (sd): 6.76 (8.53) Median (iqr): 10.67 (0; 13.64)	Mean (sd): 9.35 (12.68) Median (iqr): 9.73 (0; 15)	Mean (sd): −1.09 (8.17) Median (iqr): 1.81 (−7.14; 5.71)	Mean (sd): 0.66 (13.67) Median (iqr): 0 (0; 4.65)
**Ultrasound Findings**	**Healthy tendon**	**Casting alone**	**Tenotomy**	**Z-Plasty** **Lenghtening**
**Vascularization—N (%)**	0 (0%)	0 (0%)	1 (9.09%)	1 (11.11%)
**Hypoechoic areas—N (%)**	1 (10%)	2 (14.29%)	9 (81.82%)	7 (77.78%)
**Calcifications**	1 (7.14%)	0 (0%)	0 (0%)	0 (0%)

A positive number stands for a reduction in AP diameter changing from neutral to dorsiflexed position, while a negative number stands for an increase in AP diameter changing from neutral to dorsiflexed position.

## Data Availability

The data presented in this study are available upon request from the corresponding author. The data are not publicly available due to specific ethical and privacy considerations.
